# Molecular Mechanisms of the Phytohormone–Heat Shock Protein Pathway in Regulating Plant Thermotolerance

**DOI:** 10.3390/plants14233706

**Published:** 2025-12-04

**Authors:** Jialiang Zhang, Yanchun Zhu, Fumin Ma, Xiao Zou, Qiuxia Lan, Xiaoran Zhou, Mengxia Li, Fei Zhou, Changxi Yin, Yongjun Lin

**Affiliations:** 1 National Key Laboratory of Crop Genetic Improvement, Huazhong Agricultural University, Wuhan 430070, China; 2 MOA Key Laboratory of Crop Ecophysiology and Farming System in the Middle Reaches of the Yangtze River, College of Plant Science and Technology, Huazhong Agricultural University, Wuhan 430070, China

**Keywords:** heat shock protein, phosphorylation, phytohormones, thermotolerance, transcription

## Abstract

Heat stress caused by global climate change poses a significant threat to agricultural production. Phytohormones, as critical signaling molecules, play pivotal roles in modulating plant responses to heat stress. This review systematically summarizes the molecular mechanisms by which eight phytohormones (auxin, gibberellin, cytokinin, ethylene, abscisic acid, brassinosteroid, salicylic acid, and strigolactone) enhance plant thermotolerance through the regulation of heat shock protein (HSP) expression and function. Specifically, auxin enhances thermotolerance by inducing auxin signaling repressor (Aux/IAA) degradation to upregulate *HSP* transcription, facilitating the formation of the auxin receptor (TIR1)-HSP90 complex to stabilize TIR1, and forming the auxin exporter (PIN)-HSP22 complex to promote auxin transport. Cytokinin enhances thermotolerance by upregulating *HSP* transcription, with stronger effects in leaves than roots. Gibberellin, salicylic acid, and ethylene enhance thermotolerance primarily by activating heat shock factor (HSF) to induce *HSP* transcription. Abscisic acid and brassinosteroid improve thermotolerance by inducing *HSP* transcription and HSP phosphorylation, while strigolactone acts via D14-mediated upregulation of *HSP* transcription. These phytohormones collaboratively regulate HSPs, forming an intricate network to enhance plant thermotolerance. Deciphering these mechanisms provides a theoretical framework for developing heat-resistant crops and optimizing cultivation techniques.

## 1. Introduction

The increasing frequency of high-temperature events, driven by global warming, poses a major threat to agricultural production. According to the Intergovernmental Panel on Climate Change, global surface temperatures have risen by 0.74 °C over the past century, with the warming rate accelerating and projected to increase by 2.2–3.5 °C by the end of the century [1,2]. Heat stress severely impairs plant growth, development, yield, and quality by disrupting oxidative homeostasis, protein stability, membrane integrity, and photosynthetic efficiency [3,4,5,6,7,8,9]. Model projections suggest that without effective mitigation, each 1 °C rise in global mean temperature could reduce average yields by approximately 6.0% for wheat (*Triticum aestivum* L.), 3.2% for rice (*Oryza sativa* L.), 7.4% for maize (*Zea mays* L.), and 3.1% for soybean (*Glycine max)* [9]. Furthermore, heat stress compromises crop quality—reducing starch content in wheat and maize grains, increasing chalkiness in rice, and suppressing oil biosynthesis in soybeans [5,6,7,8].

To cope with high-temperature stress, plants have evolved sophisticated adaptive mechanisms, among which the heat shock protein (HSP) system plays a central role. As molecular chaperones, HSPs prevent protein denaturation under heat stress and assist in the refolding or degradation of misfolded proteins [10]. The transcription of *HSP* genes is primarily regulated by heat shock factors (HSFs), which bind to heat shock elements in the promoters of *HSP* genes [11]. Together, HSFs and HSPs constitute a robust protein quality control network essential for thermotolerance [10]. Notably, phytohormones intricately modulate HSP expression and function. Eight major phytohormones—auxin, gibberellin (GA), cytokinin (CK), abscisic acid (ABA), brassinosteroid (BR), salicylic acid (SA), ethylene (ETH), and strigolactone (SL)—enhance plant thermotolerance by regulating HSP expression and function through distinct signaling pathways [12,13,14].

This review systematically summarizes the molecular mechanisms through which eight phytohormone-HSP pathways enhance plant thermotolerance, focusing on how phytohormone signaling regulates HSP expression and function. These insights not only advance our understanding of plant heat stress responses, but also provide a theoretical foundation for developing innovative heat-resistant cultivation techniques and breeding thermotolerant crop varieties.

## 2. The Auxin–HSP Pathway Regulates Plant Thermotolerance

Auxins are fundamental phytohormones governing diverse aspects of plant growth and development. Their functions hinge on precise spatiotemporal regulation, achieved through a balance of biosynthesis, inactivation (via conjugation), degradation, and polar transport [15,16]. The core auxin signaling pathway operates on a unique “degrade-and-release” mechanism. In the absence of auxin, auxin signaling repressor Aux/IAA proteins bind to and inhibit AUXIN RESPONSE FACTOR (ARF) transcription factors, preventing the expression of auxin-responsive genes. When auxin is present, it binds to the TIR1/AFB family of F-box receptors, acting as a molecular glue that promotes the interaction between TIR1/AFB and the Aux/IAA repressors. This targets Aux/IAA proteins for ubiquitination and subsequent degradation by the 26S proteasome [15,17]. The removal of Aux/IAA liberates ARFs, allowing them to activate or repress downstream gene expression programs, thereby orchestrating the plant’s physiological response [15].

**Figure 1:**
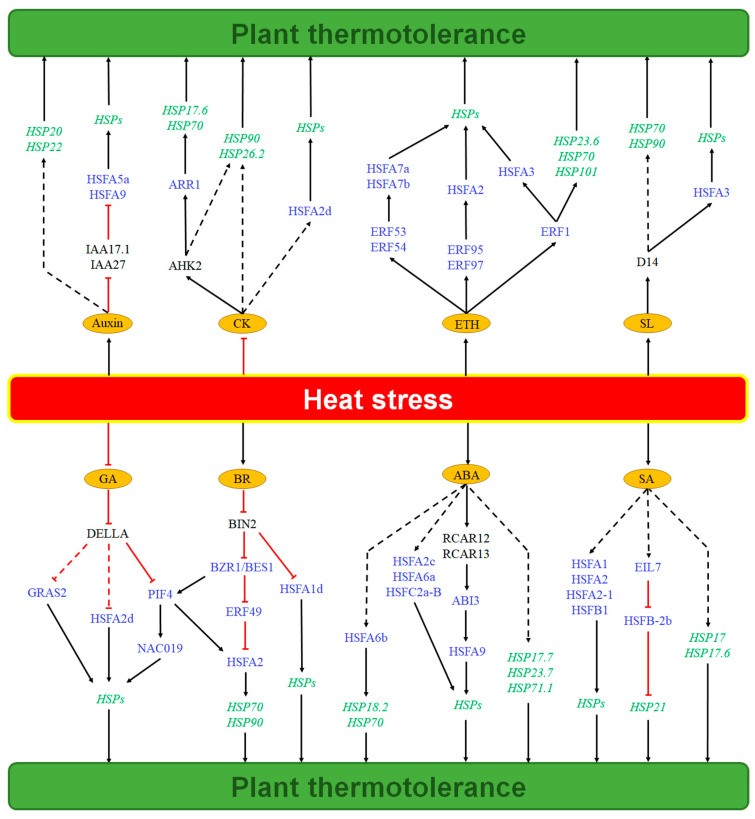
Phytohormones enhance plant thermotolerance through the regulation of *HSP* transcription. The arrows and T sharp symbol indicate positive regulation and negative regulation, respectively. Solid lines indicate established regulatory mechanisms, and dashed lines indicate unknown regulatory mechanisms. GA, gibberellin; CK, cytokinin; BR, brassinosteroid; ETH, ethylene; ABA, abscisic acid; SL, strigolactone; SA, salicylic acid; IAA17.1/27, AUX/IAA proteins (auxin signaling repressors); HSF, heat shock factor; HSP, heat shock protein; DELLA, gibberellin signaling repressor; GRAS2, gibberellin signaling transcriptional activator; PIF4, phytochrome-interacting factor4; NAC019, NAC domain containing protein19; AHK2, cytokinin receptor; ARR1, type-B ARR (cytokinin signaling transcriptional activator); BIN2, brassinosteroid signaling repressor; BZR1/BES1, the central transcription factor in brassinosteroid signaling; ERF1/53/54/95/97, ethylene signaling transcriptional activators; RCAR12/13, ABA receptors; ABI3, abscisic acid signaling transcriptional activator; D14, DWARF14 (strigolactone receptor).

Building upon this foundational framework, auxin emerges as a central regulator not only of plant growth and development but also of heat stress responses [18,19] (Figure 1). Under heat stress, plants typically exhibit increased auxin accumulation [20]. The phytohormone enhances thermotolerance primarily through transcriptional regulation of *HSPs*. For instance, auxin has been shown to promote *HSP20* transcription in pakchoi (*Brassica rapa* subsp. *chinensis*) and induce *HSP22* transcription in Arabidopsis (*Arabidopsis thaliana*); however, the specific regulatory mechanisms governing the transcription of *HSP20* and *HSP22* remain unclear [21,22]. The auxin signaling repressor Aux/IAA plays a critical role in this process by negatively regulating *HSP* transcription, likely through the inhibition of HSFs activation (Figure 1). Specific examples include PtIAA17.1 in *Populus tomentosa* and HaIAA27 in sunflower (*Helianthus annuus* L.), which suppress the transcriptional activation of HSFA5a and HSFA9, respectively [23,24]. Therefore, under heat stress, auxin-induced degradation of Aux/IAA proteins relieves this inhibition on HSFs, leading to the upregulation of *HSP* transcription and enhanced thermotolerance. However, this model of auxin-mediated regulation on HSF/HSP may not be conserved across all species. For instance, the strawberry (*Fragaria vesca*) receptacle exhibits high expression of auxin biosynthetic genes and high auxin content, yet does not significantly activate HSF or HSP expression, suggesting a species-specific adaptation [25].

**Figure 2:**
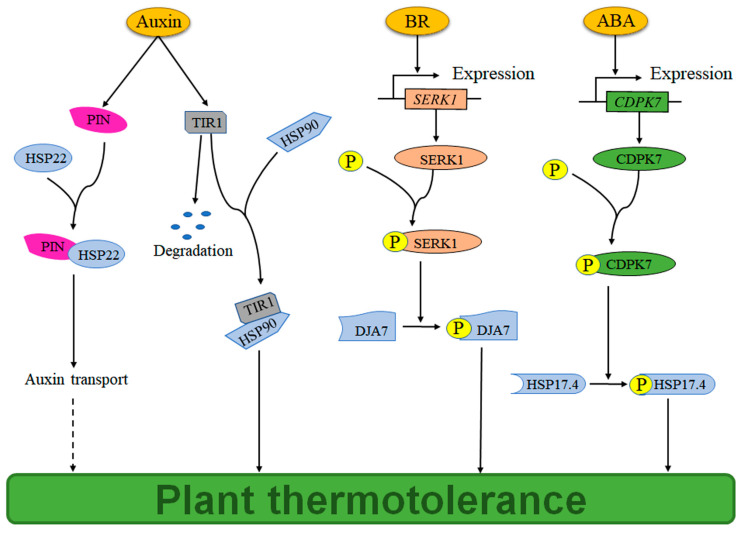
Molecular mechanisms of Auxin-, BR-, and ABA-mediated plant thermotolerance through the regulation of HSP function. Auxin enhances plant thermotolerance by facilitating auxin transport via the PIN-HSP22 protein interaction, and stabilizing the TIR1 auxin receptor through formation of a TIR1-HSP90 molecular chaperone complex. BR induces SERK1 gene transcription and protein phosphorylation, which enhances plant thermotolerance by phosphorylating DJA7. ABA induces CDPK7 gene transcription and protein phosphorylation, which enhances plant thermotolerance by phosphorylating HSP17.4. The arrows indicate positive regulation. The solid lines indicate established regulatory mechanisms, and the dashed line indicates unknown regulatory mechanisms. BR, brassinosteroid; ABA, abscisic acid; PIN, auxin exporter; TIR1, auxin receptor; SERK1, somatic embryogenesis receptor kinase1; CDPK7, calcium-dependent protein kinase 7; DJA7, a member of HSP40.

Beyond this canonical signaling pathway, auxin can further enhance thermotolerance by stabilizing its own receptor TIR1 and promoting auxin transport. Specifically, auxin facilitates the formation of a TIR1-HSP90 complex, which stabilizes the TIR1 receptor [26] (Figure 2). Concurrently, auxin promotes the formation of a complex between the auxin exporter PIN and HSP22, thereby enhancing auxin transport capacity [22] (Figure 2). These mechanisms collectively underscore the multifaceted role of auxin in fortifying plants against heat stress.

## 3. The GA-HSP Pathway Regulates Plant Thermotolerance

GAs are classical phytohormones essential for promoting key developmental processes such as seed germination, stem elongation, and flowering [27,28]. The bioactive GA levels within plants are dynamically regulated by a balance of biosynthesis, deactivation, and transport [29]. GAs are biosynthesized through a complex, multi-step pathway in plastids and the endoplasmic reticulum, leading to bioactive forms like GA_1_, GA_3_, and GA_4_ [28]. To fine-tune activity, bioactive GAs can be irreversibly deactivated through hydroxylation or conjugation. The core GA signaling pathway has been well-established. In the absence of GA, the DELLA family of transcriptional repressors (a subfamily of GRAS proteins) physically interacts with and inhibits various transcription factors, thereby restraining growth [30,31]. Upon GA perception, the hormone binds to its soluble receptor, GID1, inducing a conformational change that promotes high-affinity binding to DELLA proteins. This GA-GID1-DELLA complex is then recognized by an SCF E3 ubiquitin ligase, leading to polyubiquitination and subsequent proteasomal degradation of the DELLA repressors [30,31]. The degradation of DELLA proteins releases their inhibition on transcription factors, allowing the activation of GA-responsive gene expression programs that drive growth and development [30,31].

As a crucial phytohormone, GA has emerged as a significant regulator of plant thermotolerance, largely mediated through the transcriptional control of *HSPs* [32,33] (Figure 1). DELLA proteins, the core repressors in GA signaling, act as pivotal nodes in this heat response. GA enhances thermotolerance by inducing degradation of DELLA proteins, thereby alleviating their repression of transcription factors (TFs). In Arabidopsis, GA enhances plant thermotolerance by relieving DELLA-mediated suppression of PIF4, which subsequently upregulates *HSPs* transcription via downstream TFs such as NAC019, ultimately improving plant thermotolerance [34,35] (Figure 1). In rice, GA reduces DELLA protein abundance and upregulates the transcription of *HsfA2d*, potentially enhancing thermotolerance through improved membrane stability [33,36] (Figure 1). However, the specific regulatory mechanism by which DELLA inhibits *HsfA2d* transcription remains unclear. Additionally, in walnut (*Juglans regia* L.), GA may enhance plant thermotolerance by counteracting DELLA suppression of GRAS2—a key TF in GA signaling pathway—thereby upregulating the transcription of *HSPs* [32]. Consistent with this, overexpression of walnut *GRAS2* in Arabidopsis significantly enhances thermotolerance [32].

Interestingly, despite the thermotolerance conferred by exogenous GA application or enhanced GA signaling, endogenous bioactive GA levels generally decrease under heat stress. Moreover, the extent of this reduction can be correlated with weaker thermotolerance [33]. This suggests that the heat-induced decline in endogenous GA may act to attenuate plant thermotolerance via the GA-HSP pathway [32].

Collectively, these mechanisms constitute the GA-HSP regulatory circuit underlying plant responses to heat stress.

## 4. The CK-HSP Pathway Regulates Plant Thermotolerance

CKs are key phytohormones first discovered for their role in promoting cell division. They are central to numerous plant developmental processes, including shoot initiation, leaf expansion, nutrient mobilization, and delay of senescence [37]. Bioactive CKs, primarily isopentenyladenine, *trans*-zeatin, and their derivatives, are synthesized via the isopentenyltransferase-mediated pathway [38]. CK homeostasis is maintained through biosynthesis, reversible conjugation, and irreversible degradation catalyzed by CK oxidase/dehydrogenase [38]. The CK signaling pathway follows a multi-step phosphorelay system. It begins with perception by membrane-localized histidine kinase receptors, which autophosphorylate upon CK binding. The signal is then transferred to cytosolic histidine phosphotransfer proteins, which shuttle into the nucleus and phosphorylate type-B Arabidopsis Response Regulators (ARRs) [37]. Phosphorylated type-B ARRs activate the transcription of primary CK response genes, including type-A ARRs, which act as rapid negative feedback regulators to fine-tune the signaling output [37].

Building upon this signaling framework, CK plays a significant role in heat stress responses [39,40] (Figure 1). Its functions during heat shock are multifaceted, including upregulating *HSP* transcription [41], activating antioxidant defense systems [42], reducing lipid peroxidation [43], and stabilizing photosynthetic apparatus [44]. For example, exogenous CK application enhances rice thermotolerance by upregulating the transcription of *HSFA2d* and *HSP* genes (including *HSP90.2*, *HSP90.3* and *HSP26.2*), though the precise regulatory mechanisms warrant further investigation [45].

Consistent with a central role for CK, overexpression of CK biosynthetic genes (*IPTs*) elevates endogenous CK levels and promotes HSP70 accumulation, thereby improving plant thermotolerance [46] (Figure 1). Furthermore, mutation of the CK receptor gene *AHK2* in Arabidopsis decreases *HSP90.1* transcription and reduces thermotolerance [47], highlighting the necessity of CK perception. The signaling pathway’s positive regulators, type-B ARR transcription factors, are instrumental in this process. CK significantly upregulates the transcription of *HSP17*.6 and *HSP70* through the type-B ARR protein ARR1, thereby enhancing plant thermotolerance [48,49] (Figure 1). Consistently, the *arr1* loss-of-function mutant exhibits downregulated transcription of *HSPs* [49]. Conversely, multiple mutants of type-A ARRs (*arr3*,*4*,*5*,*6*,*8*,*9*) enhance CK signaling, upregulate *HSP* transcription, and confer stronger thermotolerance [50]. Notably, these mutants accumulate antioxidants such as α- and γ-tocopherols [50] (Figure 1), suggesting that enhanced CK signaling may mitigate heat-induced oxidative damage by boosting antioxidant capacity.

Enhanced CK signaling improves thermotolerance via the upregulation of molecular chaperones such as HSP70 and HSP90, which help stabilize the proteome under heat stress [45,51] (Figure 1). CK also induces the production of small HSPs, which protects photosystem II from heat-induced damage and alleviates heat-induced photosynthetic inhibition [43,52] (Figure 1). Importantly, CK-mediated upregulation of *HSPs* is more strongly in leaves than in roots [46] (Figure 1), likely reflecting differential thermal sensitivity across tissues.

However, despite the thermotolerance conferred by enhanced CK signaling, heat stress itself typically reduces endogenous CK accumulation. This heat-induced decline in CK levels attenuates plant thermotolerance via the CK-HSP pathway [39,45]. Together, these integrated mechanisms form the core of the CK-HSP regulatory network governing plant thermotolerance.

## 5. The ABA-HSP Pathway Regulates Plant Thermotolerance

ABA, initially discovered for its role in abscission and dormancy, is a pivotal phytohormone governing plant responses to abiotic stresses such as drought, salinity, and cold [53]. ABA levels are dynamically regulated by biosynthesis, primarily via the carotenoid pathway, and inactivation through hydroxylation and conjugation. The core ABA signaling pathway operates through a central regulatory module known as the PYR/PYL/RCAR-PP2C-SnRK2 cascade [53]. In the absence of ABA, clade A protein phosphatases 2C (PP2Cs) dephosphorylate and inhibit SNF1-related protein kinase 2s (SnRK2s) [54]. Under stress conditions, accumulated ABA is perceived by soluble receptors from the PYR/PYL/RCAR family. The hormone-receptor complex then binds to and inhibits PP2Cs, relieving their suppression of SnRK2s [54]. The activated SnRK2s subsequently phosphorylate downstream targets, including transcription factors like ABFs/AREBs, to initiate ABA-responsive gene expression, leading to stress adaptation [54].

Consistent with its role as a central stress hormone, plants typically exhibit increased ABA levels under heat stress [55]. ABA significantly enhances thermotolerance through multiple mechanisms that modulate the transcription and function of HSPs (Figure 1). As key molecular chaperones, HSP70 proteins play extensive roles in plant stress responses [56]. ABA upregulates *HSP70* transcription, and their synergistic interaction effectively mitigates heat-induced damage, thereby improving cellular thermotolerance in tobacco (*Nicotiana tabacum* L.) and maize [57,58]. This induction of *HSP70* transcription under heat stress is mediated through an extracellular H_2_O_2_-dependent pathway [57,58,59]. Similarly, exogenous ABA application enhances thermotolerance in rice seedlings by inducing the transcription of *OsHSP23.7* and *OsHSP17.7* [60] (Figure 1). However, studies on *Agrostis stolonifera* reveal a more complex regulatory mechanism, where ABA promotes *HSP17* expression in roots while inhibiting it in leaves, revealing tissue-specific differences in the ABA-HSP pathway within a single species [61].

The induction of HSPs by ABA is primarily achieved through the activation of HSFs (Figure 1). Specifically, HSFA6b acts as a positive regulator downstream of ABA signaling to activate the transcription of *HSP18.2* and *HSP70*, thereby conferring thermotolerance [62]. However, the precise mechanism by which ABA regulates *HSFA6b* transcription remains elusive. HSFA9 not only regulates *HSP* transcription but also interacts synergistically with the ABA-responsive transcription factor ABI3 to enhance thermotolerance [63]. In wheat, the transcription factor TaHsfC2a-B modulates *HSPs* transcription via the ABA signaling pathway, providing thermoprotection during grain development [64]. A similar mechanism is observed in tall fescue, where ABA upregulates *HSPs* transcription through HSFA2c [65] (Figure 1). Beyond the HSF-mediated pathway, ABA also enhances thermotolerance by upregulating the transcription of *calcium-dependent protein kinase* (*CDPK7*). The encoded protein, CDPK7, can phosphorylate sHSP17.4, further improving thermotolerance [66] (Figure 2).

The core ABA signaling pathway is directly involved in thermotolerance [67]. In Arabidopsis, overexpression of ABA receptor genes *RCAR12* or *RCAR13* enhances thermotolerance by promoting the accumulation of HSP18.2 and HSP70 [68] (Figure 1). Under heat stress, ABA rapidly upregulates the transcription of *OsHSP71.1* in rice [69]. Intriguingly, HSPs reciprocally regulate ABA signaling, forming a positive feedback loop. For example, HSPs help stabilize PYR/PYL/RCAR receptors, thereby maintaining the integrity of ABA signaling [70]. Conversely, reduced activity of HSP90.2 leads to increased ABA sensitivity [71], underscoring the importance of these feedback mechanisms in coordinating ABA signaling and heat stress responses.

## 6. The BR-HSP Pathway Regulates Plant Thermotolerance

BRs are a class of polyhydroxylated steroid hormones essential for plant growth and development, initially identified for their role in promoting cell elongation [72,73,74]. BRs regulate diverse processes, including seed germination, photomorphogenesis, and xylem differentiation [75,76]. The biosynthesis of BRs, such as the most active form brassinolide, occurs through a complex pathway starting from campesterol, with key steps involving C-6 oxidation [77]. BR homeostasis is maintained by biosynthesis and inactivation through sulfonation, glycosylation, or hydroxylation [73]. The core BR signaling pathway is initiated by perception at the cell surface by the leucine-rich repeat receptor kinase BRI1. BR binding induces heterodimerization with its co-receptor BAK1 and subsequent transphosphorylation, activating the receptor complex [78]. This activation leads to the dephosphorylation and accumulation of key transcription factors BZR1 and BES1 in the nucleus, which orchestrate the expression of BR-responsive genes [78,79]. A central negative regulator in this pathway is the glycogen synthase kinase 3-like kinase BIN2, which phosphorylates BZR1/BES1 in the absence of BR, promoting their degradation or cytoplasmic retention [78,79].

As a crucial phytosteroid hormone, BR plays vital roles in regulating plant thermotolerance, and heat stress generally increases BR levels in plants [80]. Elevated BR levels promote the degradation of the negative regulator BIN2 and enhance the transcriptional activity of the positive regulators BZR1/BES1, thereby activating the transcription of downstream BR-responsive genes. Notably, BR significantly enhances plant thermotolerance by inducing the accumulation of HSPs [81] (Figure 1).

A key mechanism involves BR alleviating BIN2-mediated suppression of HSFA1d. By inhibiting BIN2, BR enhances the DNA-binding and transcriptional activation capacity of HSFA1d, leading to the upregulation of its target *HSPs* (including *HSP90*) and consequently improving thermotolerance in Arabidopsis [82] (Figure 1). Consistently, under heat stress, Arabidopsis *BIN2* loss-of-function mutants (*bin2-3/bil1/bil2*) exhibit upregulated *HSP* transcription and enhanced thermotolerance, whereas the *BIN2* gain-of-function mutant (*bin2-1*) shows opposite effects [79,82] (Figure 1).

BR also regulates HSP expression and function through BZR1/BES1-mediated signaling. In Arabidopsis, BR signaling enhances thermotolerance through BZR1/BES1, which suppresses ERF49, thus relieving its inhibition of HSFA2 and ultimately upregulating the transcription of *HSP70* and *HSP90* [75] (Figure 1). In pepper (*Capsicum chinense*), BES1 directly binds to and activates the transcription of *HSP90* and *HSP70* genes to enhance thermotolerance [83]. Accordingly, BES1-silenced pepper plants show specific downregulation of these *HSP* genes and reduced thermotolerance [83] (Figure 1). Furthermore, BR employs a non-transcriptional mechanism to enhance thermotolerance in wheat, which involves SERK1-mediated phosphorylation and activation of the HSP40 family member TaDJA7 [84] (Figure 2).

Beyond these direct pathways, BR integrates light signaling and heat shock responses through the BZR1/BES1-PIF4 signaling hub, forming a broader thermoregulatory network [80,85] (Figure 1). Specifically, BR modulates *HSP* transcription through the BZR1/BES1-PIF4-HSFA2 transcriptional cascade, fine-tuning the plant’s adaptive response to temperature fluctuations across diverse environments [75] (Figure 1).

## 7. The SA-HSP Pathway Regulates Plant Thermotolerance

SA, a phenolic compound derived from the phenylpropanoid pathway, is a key phytohormone best known for its central role in plant defense against biotic stresses, particularly in establishing systemic acquired resistance [86]. Beyond disease resistance, SA also regulates essential physiological processes such as seed germination, stomatal closure, and photosynthesis [87,88,89]. SA biosynthesis occurs primarily via the isochorismate synthase pathway in plastids, with a minor contribution from the phenylalanine ammonia-lyase pathway [90]. SA levels are tightly controlled through biosynthesis, conjugation (mainly with glucose to form SA O-glucoside for storage), and hydroxylation for catabolism [90]. The core SA signaling pathway involves the transcriptional coactivator NPR1 [86,91]. In the absence of signal, NPR1 exists as an oligomer in the cytoplasm. Upon SA accumulation, cellular redox changes trigger NPR1 monomerization, and these monomers translocate to the nucleus. There, they interact with TGA transcription factors to activate the expression of pathogenesis-related genes and other SA-responsive genes, thereby orchestrating the plant’s immune response [86].

Building upon its well-established role as a signaling molecule, SA, a crucial phenolic phytohormone, also plays pivotal roles in heat stress responses [92]. Heat stress typically enhances SA biosynthesis and promotes its accumulation [93]. SA enhances plant thermotolerance primarily by modulating the expression and function of HSPs [94,95,96] (Figure 1).

SA regulates HSP expression largely through the activation of HSFs. In Arabidopsis heterologously expressing the wheat transcription factor gene *TaHsfA2-1*, SA significantly upregulates TaHsfA2-1 expression, which in turn activates downstream *HSP* transcription and enhances thermotolerance [97] (Figure 1). In tomato (*Solanum lycopersicum* L.), SA increases the binding activity of multiple HSFs (including HSFA1, HSFA2, and HSFB1) to the *HSP70* promoter under heat stress. This leads to elevated HSP70 protein levels, reduced heat-induced cell death and improved thermotolerance [98,99] (Figure 1). A similar protective mechanism is observed in pea (*Pisum sativum* L.), where SA promotes HSP70 accumulation to alleviate heat-induced oxidative damage. Conversely, inhibiting SA biosynthesis with 1-aminobenzotriazole blocks this effect and compromises thermotolerance [93] (Figure 1).

Beyond HSP70, SA also induces the accumulation of various small HSPs such as HSP17, HSP17.6, and chloroplast-localized HSP21, but the precise regulatory pathway controlling this process remains to be elucidated (Figure 1). These small HSPs enhance plant thermotolerance by maintaining cellular antioxidant capacity and preventing heat-induced decline in photosynthetic efficiency [93,95,100].

Furthermore, SA can enhance plant thermotolerance by upregulating the transcription of *ETHYLENE INSENSITIVE 3-like* (*EIL7*) gene (Figure 1), although the specific mechanism controlling this transcriptional upregulation has not yet been elucidated. EIL7 protein counteracts the transcriptional repression imposed by HSFB-2b on HSP21, thereby promoting its expression and improving thermotolerance in Arabidopsis [95,96].

The SA-HSP pathway does not function in isolation but interacts with other hormonal pathways. Notably, SA has been shown to increase auxin levels in rice, which works in concert with SA-induced *HSP* transcription and reduced reactive oxygen species accumulation to enhance thermotolerance [101] (Figure 1). This hormonal crosstalk underscores the complexity and integration of signaling networks in plant heat stress responses.

## 8. The ETH-HSP Pathway Regulates Plant Thermotolerance

ETH is a simple gaseous phytohormone, best-known for its effects on triple-response morphology and fruit ripening [102,103]. It regulates a diverse array of developmental processes and stress responses, including seed germination, leaf senescence, and responses to biotic and abiotic stresses [104,105]. Its biosynthesis begins with the conversion of methionine to S-adenosyl-L-methionine (SAM) by SAM synthetase, followed by the formation of 1-aminocyclopropane-1-carboxylic acid (ACC) catalyzed by ACC synthase, a key rate-limiting step [104]. ACC is then oxidized by ACC oxidase to produce ETH [106]. ETH signaling is initiated by its binding to endoplasmic reticulum-localized receptors (e.g., ETR1, ERS1) [104]. In the absence of ETH, these receptors activate the negative regulator CTR1, which suppresses downstream signaling [104,107]. However, in the presence of ETH, binding inactivates the receptors and CTR1, leading to the stabilization of the central transcription factor EIN3 and its homologs [104]. Subsequently, EIN3 activates the transcription of primary response genes, including the ETHYLENE RESPONSE FACTOR (ERF) family, which in turn regulates a vast array of secondary response genes, tailoring the plant’s physiological and adaptive outputs [107].

Consistent with its role as a stress hormone, ETH levels are generally elevated in plants under heat stress [108]. ETH enhances thermotolerance primarily by regulating the transcription of *HSPs* through a signaling cascade involving ERFs and HSFs (Figure 1). This conserved ERF-HSF-HSP regulatory module alleviates heat-induced protein denaturation and oxidative stress, thereby maintaining cellular homeostasis [12,109].

In Arabidopsis, ETH signaling enhances thermotolerance through several distinct ERF-HSF pathways: The ERF95/ERF97-HSFA2 cascade: ERF95 and ERF97 directly bind to and activate the *HSFA2*. This transcriptional activation helps stabilize cell membranes, reduce heat-induced electrolyte leakage, and ultimately improve thermotolerance [109] (Figure 1). The ERF53/54-HSFA2/7 pathway: Heat stress upregulates the transcription of *ERF53* and *ERF54*, leading to increased protein levels. The ERFs subsequently enhance the expression of HSFA2, HSFA7a, and HSFA7b, thereby improving thermotolerance [110] (Figure 1). ERF1-mediated regulation: ERF1 enhances thermotolerance by directly binding to DRE/CRT *cis*-elements in promoters of *HSFA3*, *HSP101*, *HSP70*, and *HSP23.6*, upregulating their expression [111] (Figure 1).

This regulatory mechanism is conserved across species. In tomato, ERF1 positively regulates the transcription of *HSFA2*, *HSP70*, and *HSP90*. Both ERF1 silencing and inhibition of ETH biosynthesis (using 1-methylcyclopropene) reduce the transcript levels of these *HSF* and *HSP* genes under heat stress, consequently compromising thermotolerance [112] (Figure 1).

Furthermore, ETH enhances plant thermotolerance by upregulating the expression of specific *HSFs*, including *HSFA1a* and multiple HSFA2 isoforms *HSFA2a*/*c*/*d*/*e/f*. This upregulation contributes to improved membrane stability and reduced reactive oxygen species accumulation [113] (Figure 1). However, the precise molecular mechanisms underlying ETH-mediated HSF regulation and the potential involvement of specific HSPs in these processes require further investigation.

## 9. The SL-HSP Pathway Regulates Plant Thermotolerance

SLs are a class of carotenoid-derived phytohormones initially discovered for their role in stimulating seed germination of parasitic weeds and later recognized as key signaling molecules in the establishment of arbuscular mycorrhizal symbiosis [114,115]. They are now established as crucial endogenous regulators of plant architecture, primarily inhibiting shoot branching [115]. SL biosynthesis originates from β-carotene and proceeds through a series of enzymatic steps involving carotenoid cleavage dioxygenases and subsequently cytochrome P450 enzymes (such as MAX1) [116]. The bioactive SLs can be modified or degraded enzymatically, and their homeostasis is tightly regulated [117]. The core SL signaling pathway involves perception by an α/β-hydrolase receptor D14 [118]. The binding of SL and D14 induces a conformational change in D14 that promotes interaction with the F-box protein D3 and the transcriptional repressor protein D53 [118]. This interaction triggers the ubiquitination and proteasomal degradation of the D53 repressor, thereby relieving its inhibition on downstream target genes and enabling SL-mediated transcriptional responses [118].

Building upon this framework, SLs, initially identified as regulators of plant branching and arbuscular mycorrhizal symbiosis [119,120], are now known to play significant roles in abiotic stress responses, particularly in thermotolerance [121,122]. Heat stress generally promotes the transcription of SL biosynthetic genes, leading to increased SL accumulation [123].

SL enhances plant thermotolerance primarily by activating the transcription of *HSPs* (Figure 1). In Arabidopsis, the *atmybs1* mutant provides key genetic evidence for this pathway. This mutant exhibits elevated SL levels due to the loss of AtMYBS1, a MYB-like transcription factor that normally represses the key SL biosynthetic gene *MAX1* [124]. The increased SL then activates the transcription of heat-responsive genes, including *HSFA3*, *HSP70*, and *HSP90*, through D14-mediated signaling, ultimately enhancing thermotolerance [124]. However, the molecular regulatory mechanism by which D14 regulates *HSFA3*, *HSP70*, and *HSP90* remains unclear. In addition, application of the exogenous SL analog GR24^5DS^ increases HSP70 accumulation and enhances thermotolerance in tomato [123]. SL may also upregulate *HSP* transcription in an ABA-dependent manner, although the precise mechanisms underlying this hormonal crosstalk require further elucidation [123] (Figure 1).

## 10. Conclusions and Perspectives

Heat stress poses a major threat to crop productivity by disrupting key physiological processes, including oxidative homeostasis, protein stability, membrane integrity, and photosynthesis. To cope with this challenge, plants have evolved a sophisticated defense system centered on HSPs, the expression and function of which are finely regulated by phytohormones. This review has highlighted the mechanisms by which eight major phytohormones enhance thermotolerance through the HSP network. Specifically, auxin promotes *HSP* transcription by inducing the degradation of Aux/IAA repressors, stabilizes the TIR1 receptor via the TIR1-HSP90 complex, and facilitates auxin transport through the PIN-HSP22 complex. CK upregulates *HSP* gene expression more strongly in leaves than in roots. GA, SA, and ETH primarily activate HSFs to stimulate *HSP* transcription. ABA and BR enhance thermotolerance both by upregulating *HSP* gene expression and by inducing HSP phosphorylation. SL acts through D14-dependent transcriptional upregulation of *HSPs*. Collectively, this review delineates the intricate regulatory landscape of the phytohormone-HSP module, providing a theoretical foundation and potential targets for improving crop heat stress resilience.

Despite significant progress, the specificity of phytohormone–HSP interactions in thermotolerance remains unclear. This is exemplified by the distinct regulatory profiles of key *HSPs*: *HSP70* is regulated by six phytohormones (GA, CK, BR, ETH, ABA, SL), whereas the involvement of auxin and SA is unclear. In contrast, *HSP90* transcription is modulated by four (GA, CK, BR, SL), and *HSP17.6* is solely controlled by CK and SA. However, it remains to be determined whether other HSPs also serve as convergence points for multiple phytohormones. Furthermore, although auxin, BR, and ABA are known to regulate HSP function at the protein level, it remains unclear whether the other five phytohormones employ similar post-transcriptional mechanisms. Beyond the established HSF and HSP components, identifying novel participants within this regulatory network is a crucial future direction. On the other hand, HSPs reciprocally induce hormone biosynthesis (e.g., SA) and amplify hormone signaling (e.g., GA, BR), forming positive feedback loops that synergistically enhance thermotolerance [125,126]. However, a critical yet unexplored question is which specific HSPs are capable of regulating the biosynthesis and the signaling of other phytohormones under heat stress.

Besides hormonal regulation, epigenetic regulation of HSPs also significantly contributes to plant thermotolerance. The expression of *HSP21*, *HSP22*, and *HSP17.6C*, for example, is activated through the maintenance of histone H3 lysine 4 trimethylation (H3K4me3) or the removal of H3K27me3, thereby enhancing plant thermotolerance [127,128]. However, whether other HSPs are similarly regulated by these epigenetic mechanisms to influence thermotolerance remains to be elucidated. Additionally, non-coding RNAs (e.g., miR160, miR164, tae-miR164) can regulate plant thermotolerance through transcriptional regulation of HSPs [129]. However, the underlying mechanisms of this regulation require further investigation.

The hormonal landscape itself is dynamically reshaped by heat stress. Elevated temperatures upregulate the levels of auxin [20], ABA [55], BR [80], SA [93], ETH [108], and SL [123] to activate the HSP network, while simultaneously suppressing the biosynthesis of GA and CK, thereby attenuating their respective HSP-mediated thermoprotective pathways [33,39]. However, the mechanistic underpinnings of how heat stress modulates this hormonal homeostasis are still elusive. Key unanswered questions concern the tissue-specific distribution of these hormones, the role of post-translational modifications in their signaling cascades, and the complex dynamics of interhormonal crosstalk under stress conditions.

From an application perspective, both genetic engineering and exogenous phytohormone application hold great promise. Strategies may include overexpressing positive regulators (e.g., ARR1, ERF1) or knocking out negative regulators (e.g., BIN2, DELLA proteins) using advanced gene-editing technologies. As climate change intensifies, integrating these molecular insights with innovative agronomic practices will be paramount for developing sustainable, thermotolerant cropping systems to ensure global food security.

## Data Availability

Data are contained within the article.
